# Ag Functionalized In_2_O_3_ Derived From MIL-68(In) as an Efficient Electrochemical Glucose Sensor

**DOI:** 10.3389/fchem.2022.906031

**Published:** 2022-05-09

**Authors:** Dooa Arif, Zakir Hussain, Amna Didar Abbasi, Manzar Sohail

**Affiliations:** ^1^ Department of Materials Engineering, School of Chemical and Materials Engineering (SCME), National University of Sciences & Technology (NUST), Islamabad, Pakistan; ^2^ Department of Chemistry, School of Natural Sciences (SNS), National University of Sciences & Technology (NUST), Islamabad, Pakistan

**Keywords:** glucose sensing, sensors, indium oxide, MIL-68(In), MOFs

## Abstract

In this study, Ag@In_2_O_3_ modified nickel foam (NF) was reported for its role as a non-enzymatic glucose sensor. Ag@In_2_O_3_ was prepared by a simple two-step method; preparation of a metal-organic framework (MOF) MIL-68(In) by solvothermal method, entrapment of Ag + by adding AgNO_3_ then drying it for 2 h to complete the entrapment process and subsequent calcination at 650°C for 3 h. The Ag@In_2_O_3_ modified NF was employed as a non-enzymatic glucose sensor to determine glucose concentrations in an alkaline medium. Two linear ranges were obtained from Ag@In_2_O_3_ modified electrode, i.e., 10 μM to 0.8 mM and 0.8–2.16 mM with a sensitivity of 3.31 mA mM^−1^ cm^−2^ and 1.51 mA mM^−1^ cm^−2^ respectively, with a detection limit of 0.49 µM. Ag@In_2_O_3_ modified NF exhibited high selectivity for glucose, among other interfering agents.

## Introduction

Glucose fuels our bodies to sustain everyday activities and is an essential carbohydrate, but its high concentration leads to an increased risk of heart diseases and diabetes mellitus. Hence, it is crucial to quantify the amount of glucose in the blood, and it is a need of the hour to explore rapid and efficient sensors for the detection of glucose (Nichols et al., 2013; Yang and Gao, 2019). For this purpose, multiple efforts have been made academically, commercially, and industrially to develop an effective electrochemical enzymatic glucose sensor for medical diagnosis, research, and management of pharmaceutics and food. Under mild conditions, two major natural enzymes, glucose oxidase and glucose dehydrogenase, show excellent selectivity towards glucose. They are extensively used in enzymatic glucose sensors as biological catalysts (Wang, 2008). Clark and Lyons (1962) were the first to introduce the concept of enzymatic glucose sensors (Clark and Lyons, 1962). However, enzyme-based sensors are expensive, chemically unstable, and sensitive to temperature, pH, and humidity.

Moreover, due to the difficulty in immobilization procedures of enzymes, research was then diverted to develop non-enzymatic glucose sensors having significant characteristics like stability, selectivity and sensitivity (Hwang et al., 2018). The most sensitive technique to detect glucose non-enzymatically is the electrochemical method based on electrocatalytic oxidation of glucose (Zhao et al., 2019). Fabrication of reliable, sensitive electrochemical sensors for biological analysis depends on the development of advanced electrocatalysts (Govindasamy et al., 2019). Recently, there has been growing attention towards metal-organic frameworks (MOFs) in electrochemical sensors’ area because of their unique features like tunable structure, large surface area, and adjustable aperture (Song, 2017; Li J. et al., 2019). Metal-organic frameworks constructed of metal ions and organic ligands are proven to be exceptional materials for producing metal oxides with intriguing microstructures (Cao et al., 2017). Metal oxides have a small specific surface area and are prone to agglomeration, contributing to undesirable electrochemical properties (Song et al., 2018). MOFs are used as a template to produce metal oxides. An effective strategy to increase metal oxides’ surface area and stability is chemically and thermally treating MOFs (Chu et al., 2020). Furthermore, MOFs-derived metal oxides have a larger surface area and provide a simple route for the movement of ions and electrons. Similarly, their hollow structure aids in lowering stress over the electrode material during the charge and discharge process that enhances their electrochemical activity and cycling stability, increasing their suitability for sensing and supercapacitor applications (Song et al., 2015; Wang et al., 2015; Salunkhe et al., 2017). When used as a sacrificial template for producing porous metal oxides, MOFs, their low conductivity and poor resistance against acid-alkali corrosion are atoned while showing good stability and catalytic activity (Li L. et al., 2019). Because of their porosity, MOFs provide fast access to ions and molecules during the transformation process. Therefore, the functionalization of calcined MOFs intensifies the effective surface area and catalytic response for electrochemical sensors. For these reasons, MOFs have attracted much attention as a precursor for synthesizing nanocomposite metal oxides (Stock and Biswas, 2012; Lü et al., 2014). For instance, Wang J. et al. (2020) developed and reported an isoniazid sensor based on calcined Zn/Ni-MOF, resulting in higher catalytic activity than pristine MOF. Compared to the aggregated microsphere without growth templates, metal oxides derived from MOFs have more benefits, such as sufficient active surface area and favorable kinetics (Wei et al., 2020). Indium oxide (In_2_O_3_) is a prominent n-type semiconductor with a wide bandgap (e.g., 3.55–3.75 eV). In_2_O_3_ has been frequently employed in many optic and electrical devices owing to its unique chemical properties (e.g., strong surface reactivity and high carrier conductivity) (Wang X. et al., 2020). MIL-68(In) is a type of MOF that can be easily synthesized in a non-aqueous medium using a solvothermal approach (Barthelet et al., 2004), and has emerged as a promising template for the production of porous nanostructure metal oxides (Wang et al., 2018; Zhang et al., 2019). The MILs, which are made up of metal-centered octahedra (MO_4_(OH)_2_, M = In, Ga, and Fe) and organic ligands like terephthalic acid, have a three-dimensional network structure with ultrahigh porosity (Volkringer et al., 2008). To generate metal doped In_2_O_3_ porous nanostructures, selecting MILs with distinct metal-centered octahedrons as the precursor or self-sacrificial template is a simple and promising technique. The introduction of a few impurity ions during the pyrolysis process of the MILs precursor can limit the continued growth of the new material nanoparticles, resulting in a reduction in the particle size of sensing materials and an increase in the active sites for sensing reaction.

Moreover, in addition to MOFs, various other porous materials such as metal oxide (Sheng et al., 2016), graphene (Zhan et al., 2014), and conducting polymer foams (Sun et al., 2016) are also used as electrode material, reported in the literature. To this end, Nickel Foam (NF) is an electrode with 3D porous structure and has the most significant current collecting properties. NF displays sizeable active surface area, good electrical conductivity, high flexibility and better mass transport properties, making it suitable to be used directly as an electrode material (Zhou et al., 2013). It has also been demonstrated that In_2_O_3,_ when doped with metals and metal oxides, shows increased sensitivity and selectivity, and reduced working temperature, and decreased response and recovery time resulting in improved performance of the sensors (Liang et al., 2012). Similarly, silver (Ag) has also been used as a dopant material to provide a specific adsorption site for the adsorption of oxygen and analyte molecules which could potentially help the catalytic oxidation on the surface of sensing materials by activating the analyte (Wang et al., 2014).

Taking the above considerations into account, we have synthesized MOFs-derived porous and well-ordered Indium oxide and their composite with Ag^+^ for glucose sensing. Herein, we propose the synthesis of porous In_2_O_3_ by thermal decomposition of Indium organic frameworks (InOF). Two steps route was followed to prepare the porous In_2_O_3_ and Ag@In_2_O_3,_ i.e., formation of MIL-68(In) also known as Indium Organic Frameworks (InOFs) and then its calcination at 650°C.

## Experimental

### Reagents

Materials including L (+)—Ascorbic acid and Terephthalic acid were bought from Merck KGaA, 64,271 Darmstadt Germany. Indium (III) nitrate hydrate and uric acid were purchased from SIGMA ALDRICH Co., United States. Silver nitrate was purchased from Duksan Pure Chemicals Co. Ltd. 635-1, KOREA. Dopamine hydrochloride was acquired from Solarbio. The purchased chemicals were not further purified and were used as received.

### Synthesis of MIL-68(In)

MIL-68(In) was prepared by following the protocols obtained from the literature (Volkringer et al., 2008). Briefly, (1.05 mmol, 408.2 g) indium nitrate was mixed with terephthalic acid (1.2 mmol, 200 mg), and DMF (70 mmol, 5 ml). This mixture was then shifted to Teflon-lined stainless-steel autoclave and placed in an oven at 100°C for 48 h. The resultant mixture was filtered and washed several times using DMF. After filtration, the resultant white precipitate was placed in a vacuum oven for 12 h at 80°C.

### Synthesis of Ag@In_2_O_3_

A two-step approach was used to prepare the Ag@In_2_O_3_ combination, as described in the literature (Xue et al., 2017). Briefly, 20 mg of as-synthesized MIL-68(In) was weighed accurately and ground for 10 min in a mortar, followed by adding of AgNO_3_ (5.4 × 10^−2^ mol/L) solution into the finely ground InOF powder to obtain white pulp. The white pulp was set for drying in an oven at 60°C for 2 h to complete the entrapment process. Finally, the synthesized Ag@InOF precursor was placed in the ceramic crucible in a muffle furnace for 3 h at 650°C to obtain Ag@In_2_O_3_.

### Fabrication of Ag@In_2_O_3_ on Nickel Foam

For electrode fabrication, 1 cm × 1 cm Nickel Foam (NF) was appropriately cleaned by immersing it in 3 M HCl and sonicated for 5 min. It was then washed with distilled water, sonicated again in ethanol for 5 min, and then dried at 60°C. For the slurry preparation, 2 mg of Ag@In_2_O_3_ was mixed with 300 µl ethanol and 20 µl Nafion, sonicated for 1 h. Finally, to prepare the working electrode, pre-treated NF was immersed in the slurry and oven-dried at 60 C.

### Characterization

Scanning electron microscopic analysis and Elemental mapping was carried out using SEM (JEOL JSM-6042A; Japan) while X-ray diffraction analysis was performed through (XRD, D5005 STOE and Cie GmbH Darmstadt, Germany), CuKa radiations (l = 1.5406◦A) at an angle (2θ) ranging from 10° to 80°. Th FTIR Analysis were conducted on PerkinElmer, Spectrum TM100 spectrophotometer using KBr pellets in the scan range of 400–4,000 cm^−1^.

### Electrochemical Studies

Electrochemical tests of the modified electrodes were performed using Gamry G750 electrochemical workstation. The three-electrode configuration, working electrodes were In_2_O_3_ and Ag@In_2_O_3_ coated on NF, a platinum (Pt) wire was used as counter electrode and Ag/AgCl as a reference electrode in 0.1 M NaOH for glucose sensing. Cyclic voltammetry for glucose sensing was performed at scan rates from 10 to 150 mVs ^−1^.

## Results and Discussion

### Structure Characterization

**FIGURE 1:**
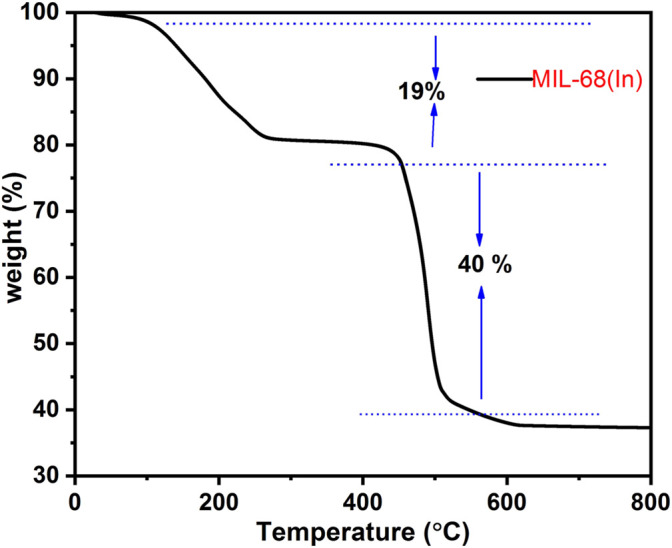
TGA analysis of MIL-68(In).

The calcination temperature has an important effect on the morphology of the MOFs-derived metal oxides. Thermo-gravimetric analysis (TGA) was performed by using a Discovery TGA 5500 TA instrument under an air atmosphere from 40 to 800°C, and the TGA curve of MIL-68 (In) is shown in Figure 1. There are two distinct stages of weight loss during the heating process. The first stage of weight loss (19%) occurs from 40 to 260°C due to the loss of adsorbed H_2_O, unreacted terephthalic acid and DMF. The second stage (40%) occurs in the range of 438–530°C. Such a large loss of weight is attributed to the oxidative decomposition of organic ligands in the MIL-68 (In) precursor. Thermal analysis reveal that the decomposition of MIL-68(In) occurs in the temperature of 400–600°C. That’s why the calcination temperature selected was above 600°C (Cui et al., 2018; Sun et al., 2021).

### Analytical Characterization

**FIGURE 2:**
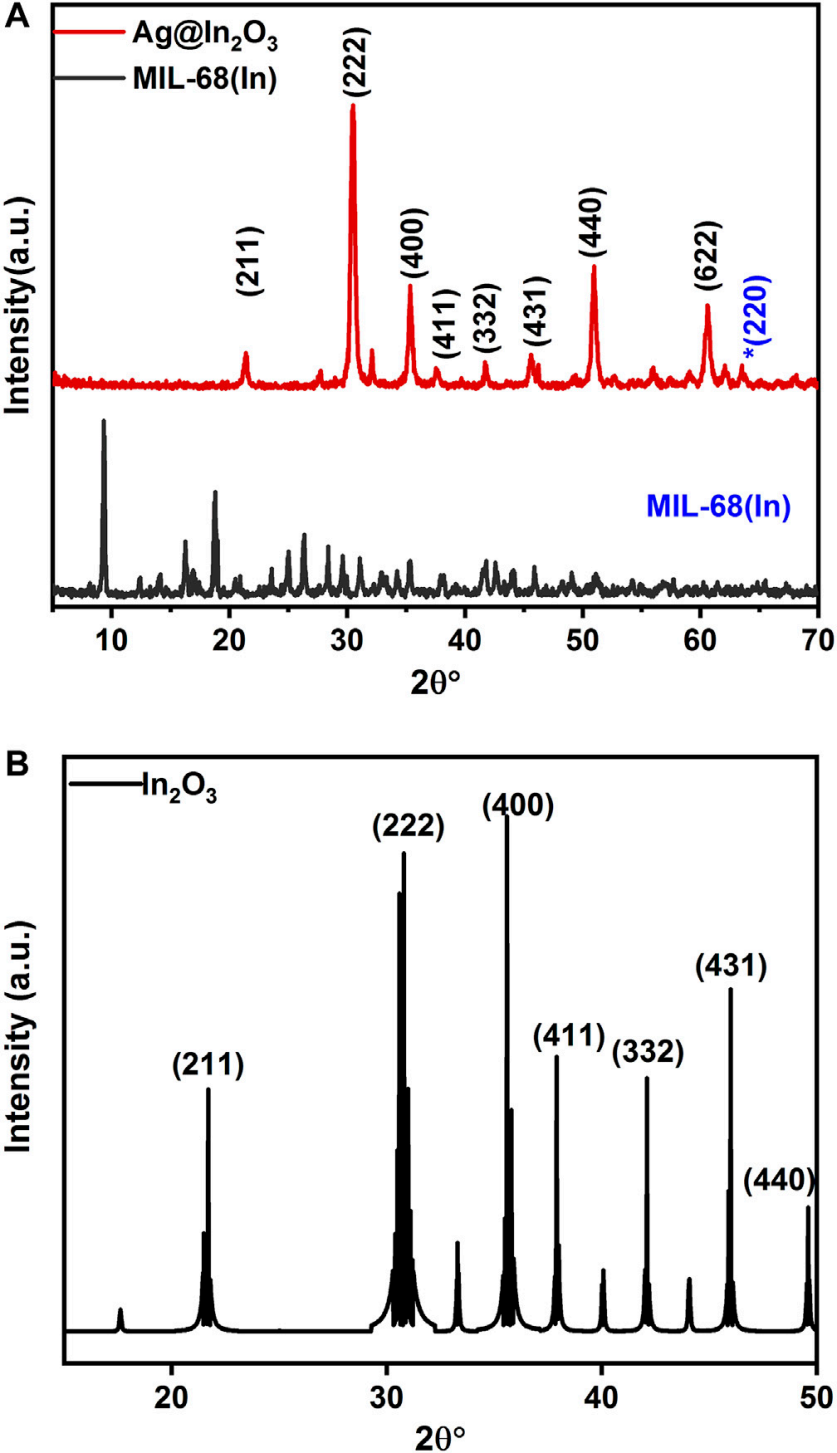
XRD pattern of **(A)** is MIL-68(In) and Ag@In_2_O_3_ whereas **(B)** is _2_O_3_.

XRD analysis of Indium organic frameworks [MIL-68(In)] shows a high degree of crystallinity Figure 2A while diffraction peaks of Ag@In_2_O_3_ are in perfect alignment with the JCPDS card No.06-0416. The 2θ angles at 21.4°, 30.5°, 35.4°, 37.6°, 42.6°, 45.7°, 50.1°, and 60.6° corresponds to (211), (222), (400), (411), (332), (431), (440), and (622) respectively (Tan et al., 2015). It is pertinent to mention that diffraction peaks for Ag in the XRD pattern are capped by peaks of In_2_O_3_ due to low weight percentage. The prominent peaks of Ag at 38° (111) (Mohammadzadeh Kakhki et al., 2019) and 44° (200) are overlapped, although a minor peak at 64° which corresponds to (220) is observed highlighted in Figures 2, XRD of the In_2_O_3_. All intense peaks in the spectrum can be well indexed to cubic In_2_O_3_ (JCPDS Card No. 06-0416, space group Ia3 (206), a = 10.118 Å) (Kim et al., 2011; Prasad et al., 2021).

**FIGURE 3:**
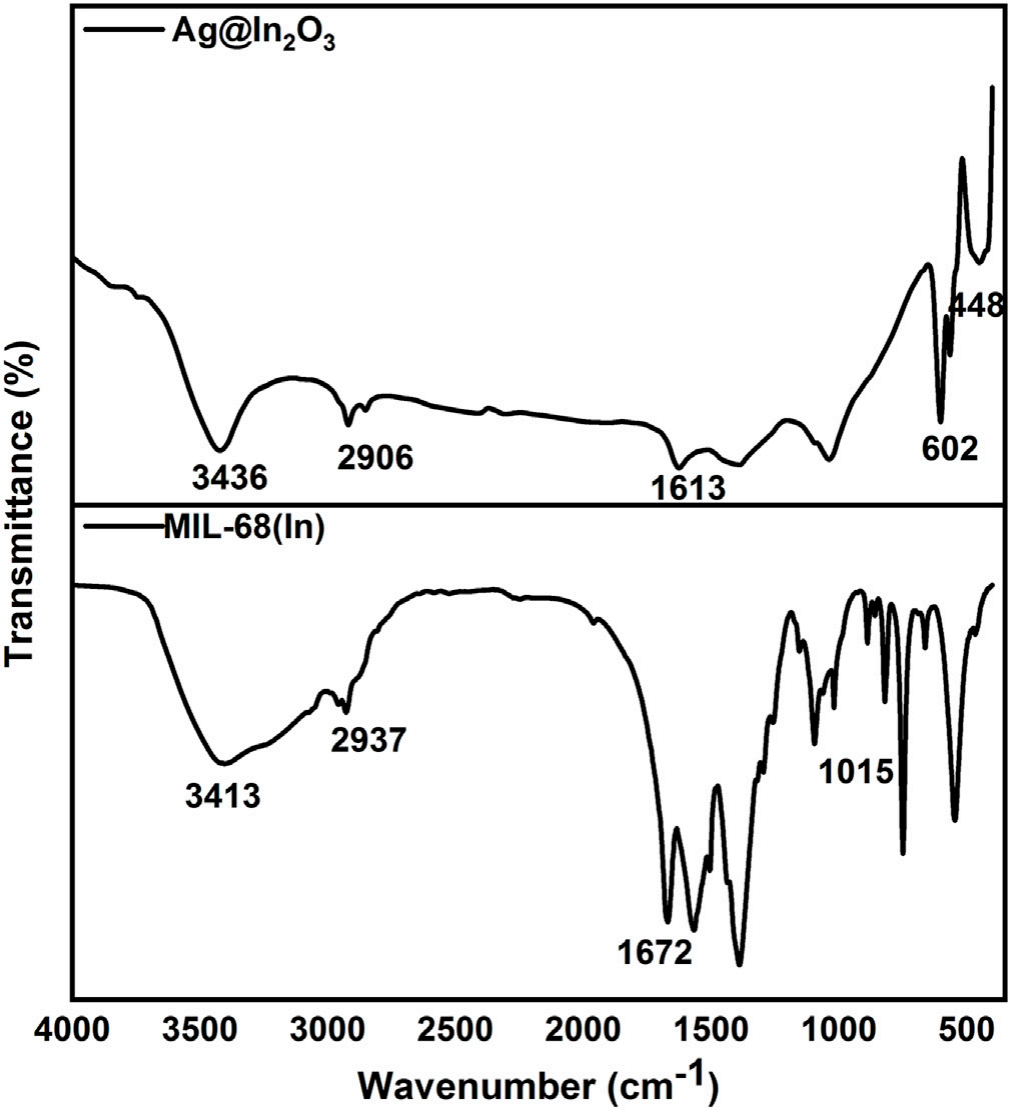
FTIR-spectra of MIL-68(In) and Ag@In_2_O_3._

**FIGURE 4:**
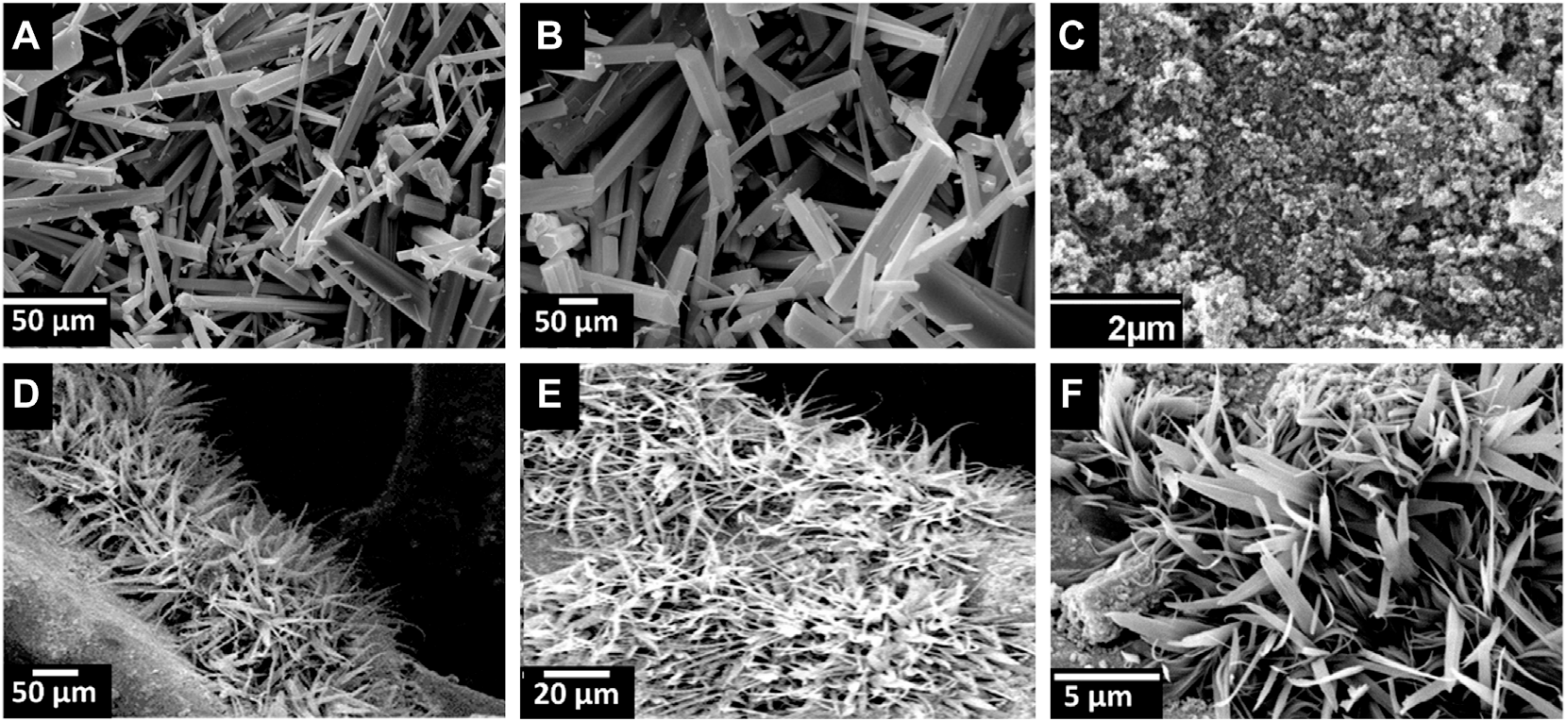
SEM micrographs of MIL-68(In) is shown in **(A) and (B)**. Figure 4**(C)** is Ag@In_2_O_3_ and **(D–F)** shows Ag@In_2_O_3_ coated on NF.

The molecular structure of MIL-68(In)-X has been investigated by FTIR measurement. As displayed in Figure 3 MIL-68(In) absorption bands are related to the vibrations of the organic unit (terephthalate). Bands ranging from 700 to 900 cm^−1^ are associated with benzene ring out of plane bending. The in plane bending of the benzene ring is shown by the peak at 1,015 cm^−1^. Peaks near 1,400 and 1,600 cm^−1^ represent the symmetric and asymmetric stretching modes of carboxylate structures attached to Indium centers, respectively. (Hu et al., 2015). In Ag@In_2_O_3_, the absorption bands in the range of 448–600 cm^−1^ shows the characteristic peaks of In-O phonon vibrations of cubic phase In_2_O_3_ (Almontasser and Parveen, 2020). 3,436 cm^−1^ shows OH stretching and 1,613 cm^−1^ are assigned to nitrate group and shows bend deformation of water (Kulkarni and Patil, 2016). For morphological characterization of the samples, scanning electron microscopy (SEM) was performed, which indicates rod-shaped crystals with smooth surface of uncalcined MIL-68(In) Figures 4A,B. Because their three-dimensional networks depict a Kagom’e-like lattice with endless chains of octahedral units linked through the terephthalate ligand delimiting triangular and hexagonal channels, hexagonal symmetrical morphologies can be seen in every image. (Cui et al., 2018). Figure 4C shows the products obtained after the pyrolysis of MOF at 650°C. It can be observed that MIL-68(In) converted into its derived metal oxide. The loss of regular shape of the single crystal after annealing should be caused by grinding process (Xue et al., 2017).

**FIGURE 5:**
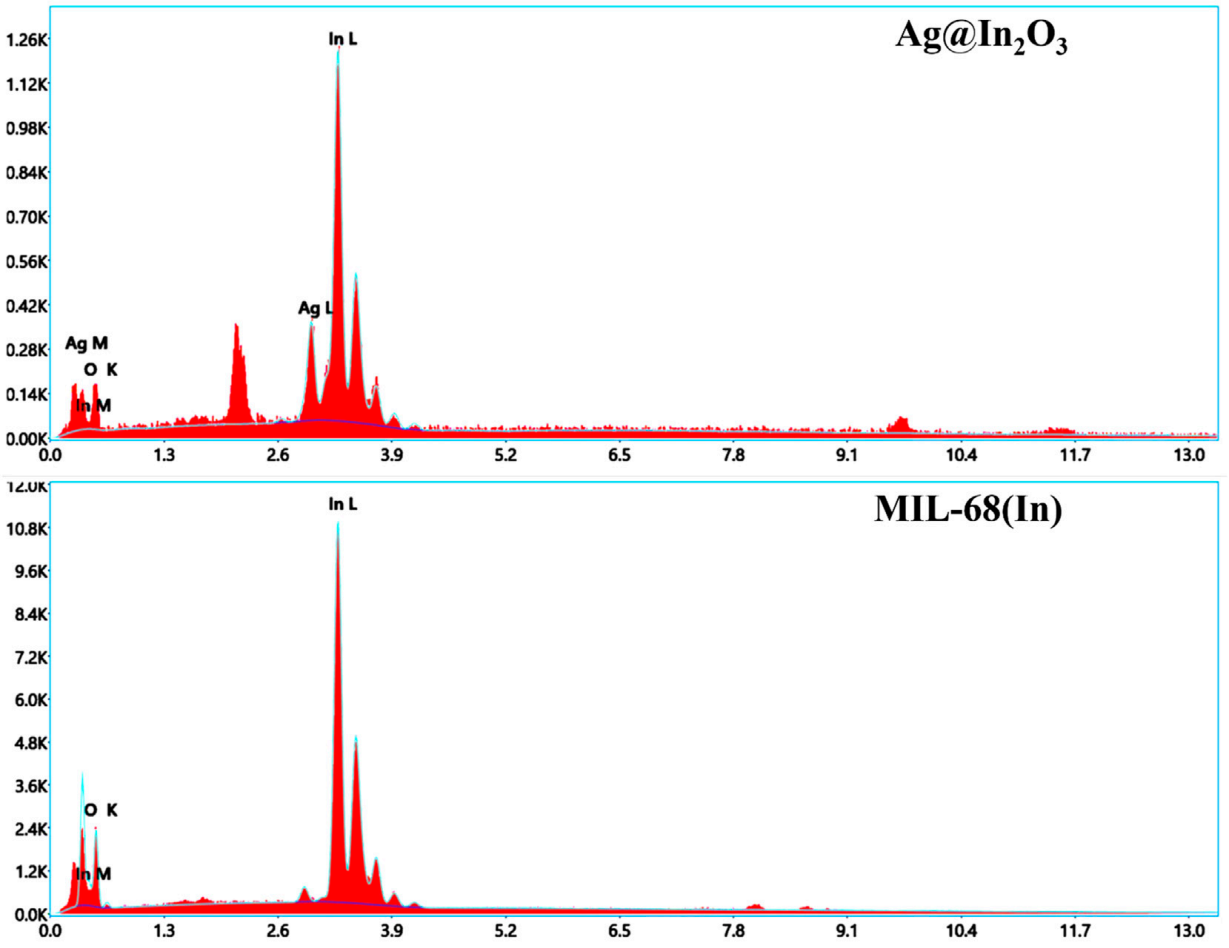
Edx analysis of MIL-68(In) and Ag@In_2_O_3._

Furthermore, the morphology of Ag@In_2_O_3_ indicates its homogeneous coverage over the surface of Ni-foam Figures 4D–F. The existence of Ag particles was proved by the EDX patterns in Figure 5.

### Cyclic Voltammetric Studies

**FIGURE 6:**
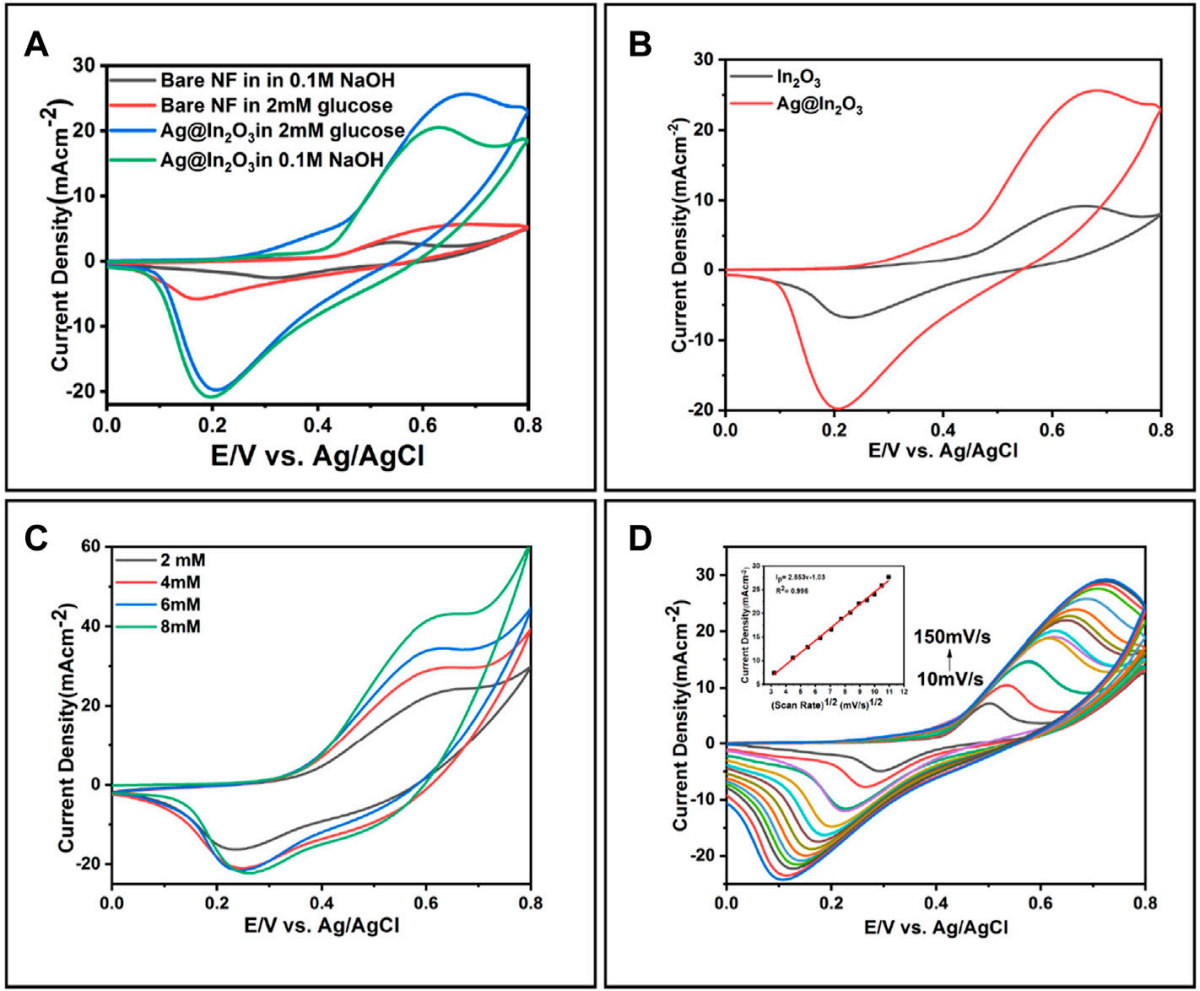
**(A)** Cyclic Voltammetry of bare NF and Ag@In_2_O_3_ in 0.1 M NaOH, with and without glucose **(B)** Comparison between the CVs of In_2_O_3_ and Ag@In_2_O_3_ in 2 mM glucose, **(C)** Response of Ag@In_2_O_3_ modified NF after successive addition of 2–8 mM glucose, **(D)** CVs of Ag@In_2_O_3_ in the presence of 2 mM glucose in the potential range of 0 V to +0.8 V at different scan rates of 10–150 mVs^−1^ (Inset: the plot of Ip vs. v).

Cyclic Voltammetry was used to determine the modified electrode’s electrochemical behavior toward glucose in the potential range of 0 to + 0.8 V. Figure 6A shows cyclic voltammograms of bare Ni-foam and Ag@In_2_O_3_ with and without glucose at a scan rate of 50 mVs^−1^. In Figure 6A, bare Ni-foam shows no response in the absence of glucose, but a small peak of 4.5 mA was observed at + 0.55 V in the presence of glucose.

A pair of redox peaks observed in CV curves suggest the presence of reversible Faradaic reaction of Ni^2+^/Ni^3+^ with the assistance of $OH^{-}$ (Wang et al., 2002). Although bare NF showed electro-oxidation towards glucose, responses are weak. However, when glucose is present, Ag@In_2_O_3_ modified electrode showed a peak at 25.2 mA at + 0.65 V. To demonstrate the effect of composite, In_2_O_3_ and Ag@In_2_O_3_ were compared in Figure 6B, and it is evident from the figure that entrapment of Ag in metal oxide increase its catalytic activity. A visible difference in the peaks currents for both electrodes can be observed. A relatively high current response of 25.6 mA at + 0.68 V was achieved by Ag@In_2_O_3_ modified electrode compared to In_2_O_3_ which showed peak current of 9.2 mA at + 0.66 V. This improved performance is the result of the perfect combination of mesoporous structure of metal oxide and Ag and their synergistic effect for the catalytic behavior. The behavior of Ag@In_2_O_3_ modified electrode against various glucose concentrations is shown in Figure 6C. This can be easily seen that by increasing the concentration of glucose from 2 to 8 mM, oxidation current increases, indicating a superior electrocatalytic behavior of Ag@In_2_O_3_ modified NF. However, after 8 mM concentration of glucose, a constant response was observed, indicating the saturation state of analyte. Investigation of the effect of potential scan rate in 0.1 M NaOH solution containing 2 mM glucose ranging from 10 to 150 mVs^−1^ shows the mechanism involved in the electrochemical process on electrode surface. Figure 6D shows the scan rate on the glucose oxidation for Ag@In_2_O_3_. With the increase in scan rate, the glucose oxidation current is increased along with a shift of anodic peaks towards positive range of the potential window +0.49 to +0.75 V, while cathodic peaks shifted towards negative potential from 0.28 to 0.09 V. This behavior suggests that the process for glucose oxidation is diffusion controlled. It can further be postulated that metal oxide (In_2_O_3_) oxidizes glucose into the gluconic acid and reduced to its lower oxidation state (In_2_O) followed by its reaction with the alkaline electrolyte (hydroxyl ion) and conversion back to In_2_O_3_ and water. This oxidation process of glucose is enhanced by the synergistic effect of Ag located nearby metal oxide. Inset of Figure 6D depicts a linearity graph between anodic peak potential and the square root of the scan rates, which indicates the kinetic limitation of the reaction between redox sites of the Ag@In_2_O_3_ modified electrode and the analyte (Liu et al., 2017).

### Amperometric Studies

For amperometric studies, first step is to analyze the optimized potential because the detection potential strongly influences amperommetric response of biosensors. Chronoamperometry determines sensitivity, response, and linear range of the modified electrodes.

**FIGURE 7:**
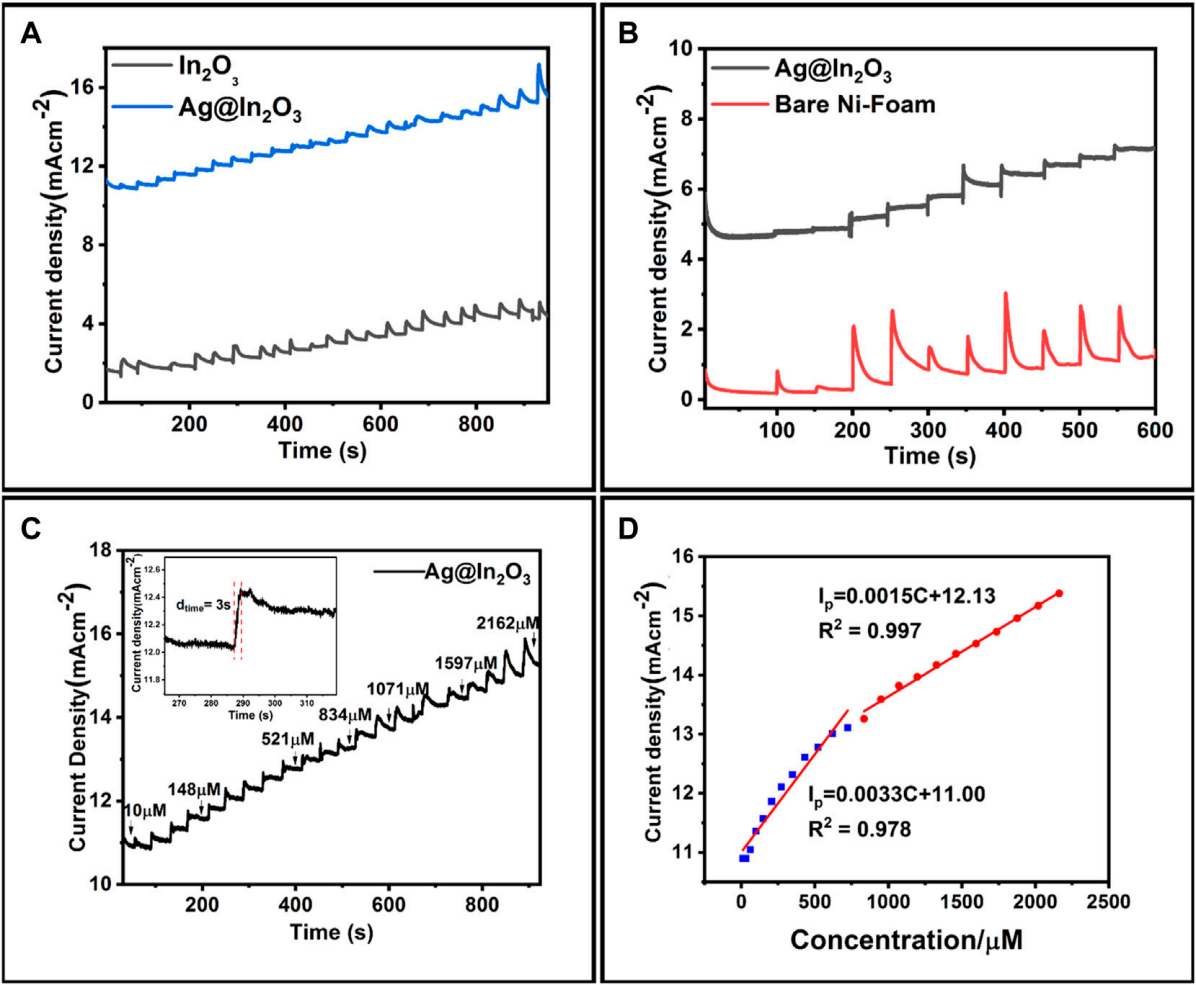
**(A)** Amperograms of Bare NF and Ag@In_2_O_3_ in NaOH (0.1 M) stirred solution with different glucose concentrations, **(B)** Amperograms of In_2_O_3_ and Ag@In_2_O_3_, **(C)** Amperogram of Ag@In_2_O_3_ modified NF at +0.7 V obtained from the stepwise additions of various glucose concentrations (30 µl after every 40 s interval) in the range of 10–2200 µM (inset: magnified amperogram showing current response of 3 s), **(D)** Two linear ranges at +0.7 V of calibration curve between glucose concentration and current response.

Figure 7A shows *i*-t curve for bare NF and Ag@In_2_O_3_ modified NF. It is evident from the results that current responses obtained for Ag@In_2_O_3_ modified electrode were prominent while no stepwise linear increase in the current responses could be observed for bare NF. This is in line with the CV graphs. Similarly, Figure 7B depicts the comparison between Ag@In_2_O_3_ and In_2_O_3_ modified electrodes and it is observed that the response of oxidation currents in In_2_O_3_ modified electrode is less as compared to Ag@In_2_O_3._ The initial oxidation current response of In_2_O_3_ was 1.9 mA when 10 µM glucose was added. Figure 7C shows the amperogram of Ag@In_2_O_3_ modified electrode obtained by adding various known glucose concentrations into the stirring solution of 0.1 M NaOH at the potential of +0.7 V. The initial oxidation current response of 10.9 mA was achieved by adding 10 µM glucose. The oxidation current of the glucose linearly increased with the increasing glucose concentrations, giving broad linear range from 10 to 2,162 µM with a correlation coefficient of 0.978 and 0.997. Response time of the sensing material against the analyte concentration is crucial for assessing the performance of an electrochemical sensor. Figure 7C, inset shows the amplified view of the response time attained upon analyte addition. It can be seen that after adding glucose, the current reached its steady state within 3 s.

Better performance of Ag@In_2_O_3_ modified electrode could be associated with increased surface area, better catalytic activity, and high conductivity as a result of synergistic effect of Ag and Indium oxide in a nanocomposite. In response to various glucose concentrations, Figure 7D shows two wide linear ranges of oxidative currents. One region is from 10 μM to 0.8 mM and the other range is from 0.8 to 2.16 mM. From Figure 7D, for 10 μM to 0.8 mM range: I_p_ = 0.0033C + 11.0 (R^2^ = 0.978)

While for 0.8–2.16 mM range: I_p_ = 0.00155C + 12.13 (R^2^ = 0.997)

The sensitivity values obtained from Ag@In_2_O_3_ modified electrode are 3.31 mA mM^−1^ cm^−2^ and 1.51 mA mM^−1^ cm^−2^ from first and second linear ranges, respectively. The limit of detection calculation is based on the signal to noise ratio (S/N = 3). We could also state that the lower detection limit is an experimental value, reflecting the minimal amount of glucose addition required to elicit a response from the electrode. The calculated detection limit is 0.49 µM by using the formula LOD = (3x noise current density)/sensitivity. The limit of quantification is 1.617 µM (Dayakar et al., 2018).

The possible explanation behind the difference in two slopes of the calibration curve is the absorption of intermediates produced during the glucose oxidation on the electrode surface (Luo et al., 2012). Furthermore, after successive administrations of glucose at high concentrations, a minor baseline drift in the amperogram is detected, which could be related to slight variations in local pH, faster glucose consumption than its diffusion, or the adsorption of intermediates on the active sites (Chen et al., 2014).

Table 1 compares our Ag@In_2_O_3_ modified electrode with other metal oxides based electrochemical sensors used for glucose sensing in the literature.

**TABLE 1:** Comparison of modified electrode with other metal oxides based electrochemical sensors for glucose sensing.

Year	Electrode composition	Sensitivity mA mM^−1^ cm^−2^	Linear range	Detection limit	References
2020	Co_3_(BTC)_2_ MOFs/GCE	1.792 and 1.002	1 μM to 0.33 mM and 0.33–1.38 mM	0.33 μM	Shahrokhian et al. (2020)
2020	Co doped Cu-MOF/Cu_2_O Nanorods/Cu foam	0.0119	0.001–1.07 mM	0.72 μM	Wei et al. (2020)
2019	Co-ZIF/NF	2.981	10–120 mM	0.42 mM	Arul and John, (2019)
2019	Co-MOF/NF	10.886	0.001–3 mM	1.3 nM	Li et al. (2019c)
2017	Ni@C/Ni foam	32.79	0.15uM—1.48 mM	50 nM	Zhang et al. (2017)
**2021**	**Ag@In**_**2**_ **O**_**3**_ **/NF**	**1.51 and 3.31**	**10 μM to 0.8** **mM and 0.8** **mM to 2.16 mM**	**0.49 μM**	**This work**

Bold values mention the values extracted from the current work.

### Interference Study

**FIGURE 8:**
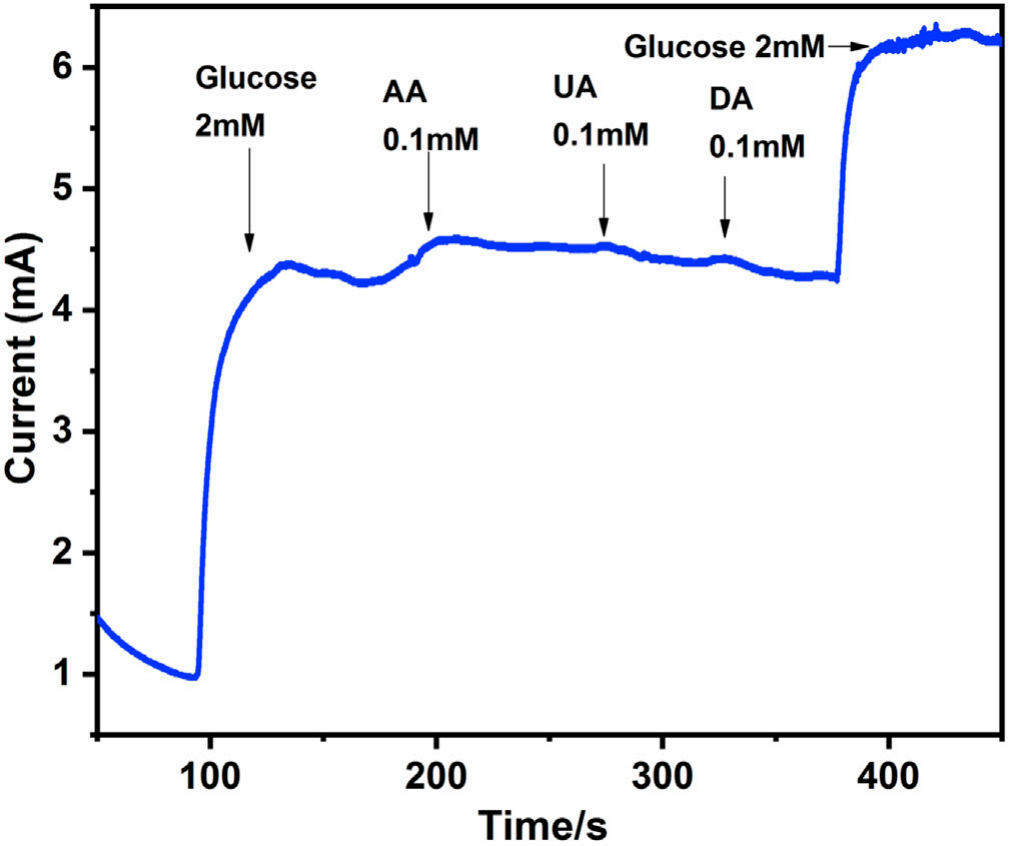
Current responses of Ag@In_2_O_3_ modified NF to 2 mM glucose, 0.1 mM of Ascorbic Acid (AA), Uric Acid (UA) and Dopamine (DA).

Another crucial analytical factor determined by amperometric experiments is differentiating glucose in blood among other electroactive species. Glucose concentration in blood lies between 4–7 mM, depending on the physiological condition of the person (Arif et al., 2020). Certain interfering species are present along with glucose in the blood with 30–50 times less concentration than glucose, but their presence can influence glucose detection. Therefore, it is essential to assess the sensor’s selectivity towards glucose (El Khatib and Abdel Hameed, 2011). Interfering agents that commonly co-exist with glucose in biological systems are uric acid (UA), dopamine (DA) and acetaminophen (AP). Figure 8 shows the response curve of the selectivity of modified electrode towards glucose. When glucose was added into 0.1 M NaOH solution, a visible peak current appeared while minimum response was shown when 0.1 mM concentration of interfering agents such as AA, UA and DA were introduced in the system. The response curve signifies that Ag@In_2_O_3_ modified electrode has good selectivity towards glucose.

### Stability and Reproducibility

Electrochemical sensor reproducibility and stability are essential parameters for practical applications. Similarly, the amperometric responses from Ag@In_2_O_3_ NF five electrodes had an RSD of 2.47%, which was satisfactory. The current response to 30 µM glucose was tested for 5 days to investigate the stability of the Ag@In_2_O_3_ NF electrode after storage at ambient temperature in air. A 3.45% RSD value was achieved.

## Conclusion

In the present investigation, an electrochemical sensor based on Ag decorated metal oxide frameworks to determine the electrocatalytic activity of glucose is demonstrated. The Ag@In_2_O_3_ showed enhanced activity compared to the pure metal oxide because of more electroactive sites, large surface to volume ratio, and fast catalytic activity because of the electrical conductivity provided by Ag^+^ in the nanocomposite. The as prepared sensor showed remarkable sensitivity values 3.31 and 1.51 mA mM^−1^ cm^−2^ for the two linear ranges of 10 μM—0.8 mM and 0.8–2.16 mM, with response time of 3 s and 0.49 µM detection limit.

## Data Availability

The original contributions presented in the study are included in the article/Supplementary Material, further inquiries can be directed to the corresponding author.
